# Assessing plant performance in the Enviratron

**DOI:** 10.1186/s13007-019-0504-y

**Published:** 2019-10-23

**Authors:** Yin Bao, Scott Zarecor, Dylan Shah, Taylor Tuel, Darwin A. Campbell, Antony V. E. Chapman, David Imberti, Daniel Kiekhaefer, Henry Imberti, Thomas Lübberstedt, Yanhai Yin, Dan Nettleton, Carolyn J. Lawrence-Dill, Steven A. Whitham, Lie Tang, Stephen H. Howell

**Affiliations:** 1 Department of Agricultural and Biosystems Engineering, Iowa State University, Ames, IA USA; 2 Present Address: Department of Biosystems Engineering, Auburn University, 213 Corley Building, 350 Mell St, Auburn, AL 36830 USA; 3 Department of Genetics, Development and Cell Biology, Iowa State University, Ames, IA USA; 4 Present Address: The Faboratory, Yale University, 9 Hillhouse Ave, ML 118, New Haven, CT 06511 USA; 5 Department of Plant Pathology and Microbiology, Iowa State University, Ames, IA USA; 6 Percival Scientific, Inc, Perry, IA USA; 7 Department of Agronomy, Iowa State University, Ames, IA USA; 8 Department of Statistics, Iowa State University, Ames, IA USA

**Keywords:** Environment, Climate change, Crop plants, Growth chambers, Robot, Hyperspectral imaging, PAM-fluorometry

## Abstract

**Background:**

Assessing the impact of the environment on plant performance requires growing plants under controlled environmental conditions. Plant phenotypes are a product of genotype × environment (G × E), and the Enviratron at Iowa State University is a facility for testing under controlled conditions the effects of the environment on plant growth and development. Crop plants (including maize) can be grown to maturity in the Enviratron, and the performance of plants under different environmental conditions can be monitored 24 h per day, 7 days per week throughout the growth cycle.

**Results:**

The Enviratron is an array of custom-designed plant growth chambers that simulate different environmental conditions coupled with precise sensor-based phenotypic measurements carried out by a robotic rover. The rover has workflow instructions to periodically visit plants growing in the different chambers where it measures various growth and physiological parameters. The rover consists of an unmanned ground vehicle, an industrial robotic arm and an array of sensors including RGB, visible and near infrared (VNIR) hyperspectral, thermal, and time-of-flight (ToF) cameras, laser profilometer and pulse-amplitude modulated (PAM) fluorometer. The sensors are autonomously positioned for detecting leaves in the plant canopy, collecting various physiological measurements based on computer vision algorithms and planning motion via “eye-in-hand” movement control of the robotic arm. In particular, the automated leaf probing function that allows the precise placement of sensor probes on leaf surfaces presents a unique advantage of the Enviratron system over other types of plant phenotyping systems.

**Conclusions:**

The Enviratron offers a new level of control over plant growth parameters and optimizes positioning and timing of sensor-based phenotypic measurements. Plant phenotypes in the Enviratron are measured in situ—in that the rover takes sensors to the plants rather than moving plants to the sensors.

## Background

Understanding the impact of climate change on plants in the environment and in cropping systems is a pressing need given that the global food supply must be doubled by 2050 to feed the planet’s burgeoning population [1]. The evidence for climate change is overwhelming, and the Fourth National Climate Change Assessment is a call for action [2]. These circumstances demand measures to abate human contributions to climate change and require that we develop means to adapt the plants we rely upon to climate change. The Enviratron facility at Iowa State University aims to address issues of the changing environment by assessing plant performance under different environmental conditions, including predicted future environments. The facility is designed to help scientists study how plants respond to different environmental conditions, so that plant performance can be adapted by design to future climate changes.

A number of plant phenotyping facilities have been developed around the world. A recent review listed selected phenotyping facilities and platforms [3], and most are high-throughput facilities designed to monitor plants non-invasively under controlled environmental conditions. The monitoring capabilities include the use of digital RGB imaging, infrared thermography, fluorescent spectroscopy, holography, spectral reflectance, magnetic resonance imaging and positron emission tomography [3]. Phenotyping under controlled conditions has allowed investigators to assess plant traits under conditions that cannot be reliably obtained in the field. However, most facilities do not allow for investigators to assess the effects of different environmental conditions.

**Fig. 1:**
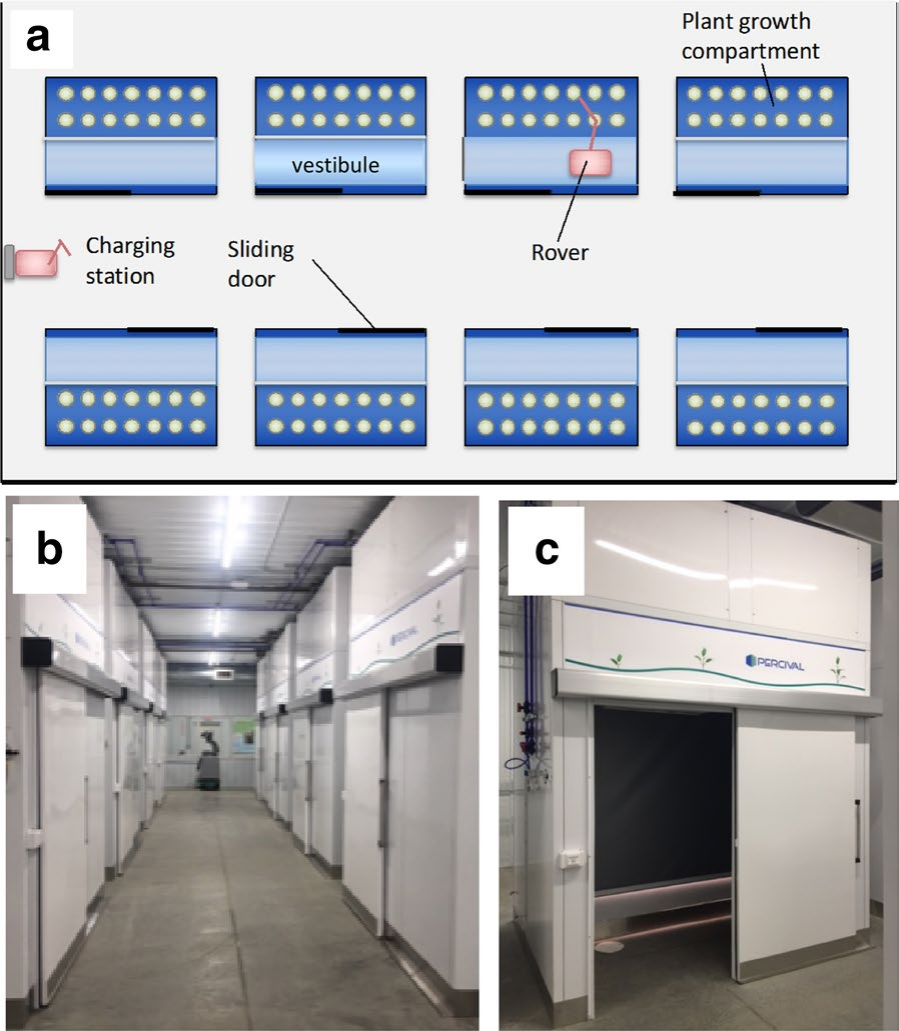
Growth chambers in the Enviratron. **a**, **b** Array of eight growth chambers in the Enviratron. Each chamber can be set to different environmental conditions. A single rover moves from chamber to chamber to monitor plant performance. Each chamber has a plant growth compartment and a vestibule to accommodate the rover. **c** Growth chamber with sliding door open into the vestibule. Once the rover is inside the vestibule, a curtain separating the vestibule from the plant compartment raises to allow the rover access to the plants

In contrast to other plant phenotyping facilities, the Enviratron enables experimenters to simulate different environmental conditions and to monitor plants nondestructively under those conditions. The Enviratron consists of an array of plant growth chambers and a roving robot (referred to as the rover) that travels from chamber to chamber to monitor plants (Fig. 1a–c). A limitation of such a facility that offers many different environments is space. So unlike high throughput facilities, the Enviratron can handle only a few genotypes at a time. Because of this, the Enviratron depends on investigators using multitier screening, in which earlier rounds of screening are conducted in the field or in high throughput phenotyping facilities [4]. In this way the Enviratron functions as a plant performance facility to evaluate the performance of selected genotypes under different environmental conditions. On the other hand, a benefit of the Enviratron is that it operates in a sensor-to-plants mode in that plants are not moved to be analyzed, instead the analyzer comes to them. In addition, because plants are not moved, large plants such as maize can be grown and monitored to reproductive maturity. Thus, the Enviratron plays an important, but largely unfulfilled role in phenotyping in that phenotype is the product of genotype and environment, and the Enviratron provides a unique platform for evaluating the effect of the environment on plant phenotype.

## Results

### Rover

**Fig. 2:**
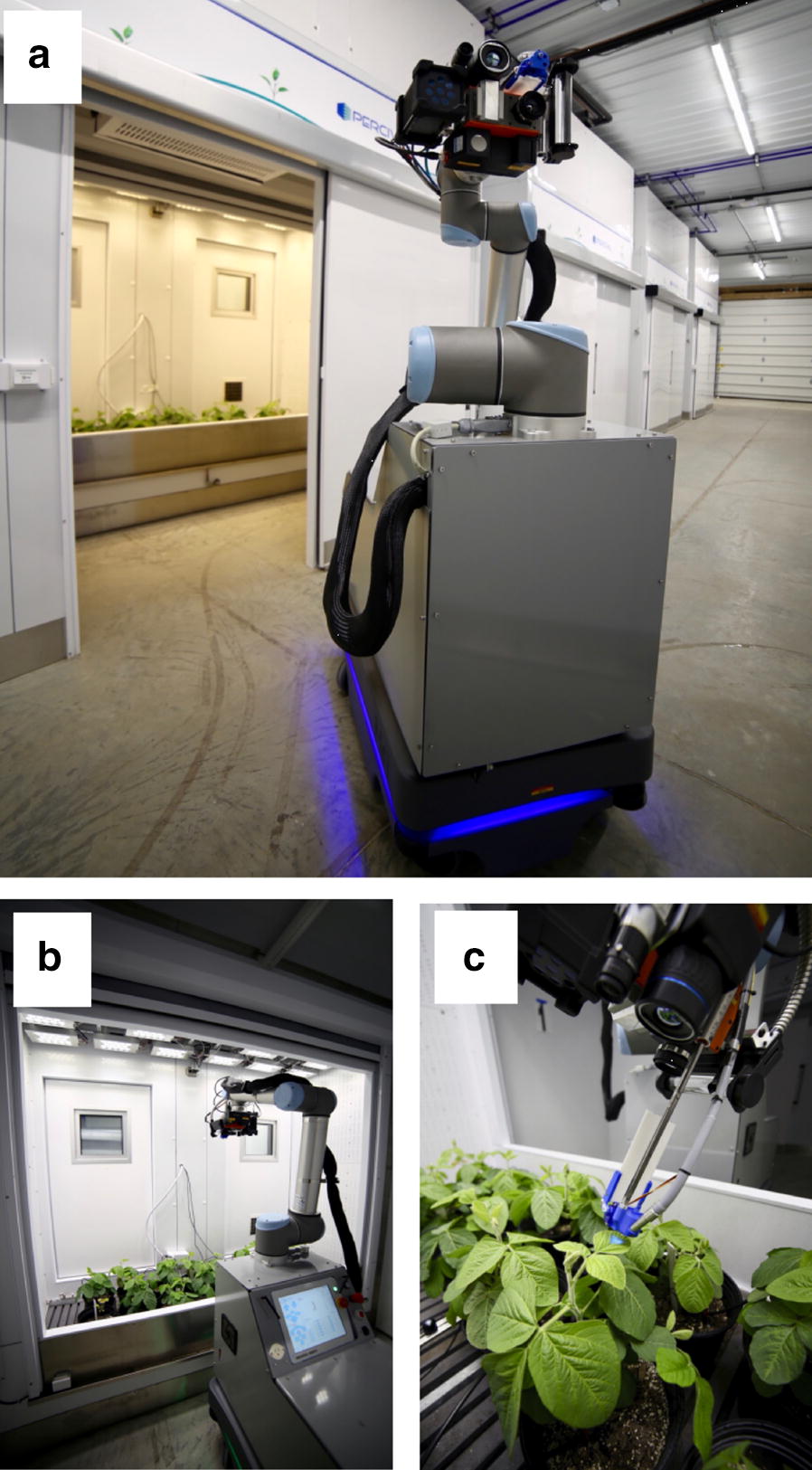
The robotic rover. **a** The rover consists of three modules from the bottom up: an unmanned ground vehicle, a six-axis robotic arm and a sensing unit at the end of the arm. **b** Operation of the robot and its robotic arm within a chamber. Sensing unit is positioned to take an overhead shot. **c** A pneumatic cylinder positions the PAM-fluorometer probe at a precise distance and angle from the leaf surface

Monitoring plants in the different chambers is enabled by the operation of a custom-built rover. The rover consists of three modules: an unmanned ground vehicle (UGV), a robotic arm, and a sensing unit (Fig. 2a). The rover shares many features with self-driving cars. The UGV is a MiR200 mobile robot (Odense, Denmark) that uses Simultaneous Localization and Mapping (SLAM) technology with two SICK S300 laser scanners (Waldkirch, Germany) for autonomous navigation in the facility [5]. Mounted on the UGV is a six-axis robotic arm with a head piece bedecked with an array of sensors. The robotic arm is a Universal Robots UR10 (Odense, Denmark) with a reach radius of 1.3 meters. The arm can be positioned to take overhead or side view shots (Fig. 2b) or to precisely project a probe on a pneumatic cylinder toward the leaf surface (Fig. 2c).

#### “Eye-in-hand” operation of the robotic manipulator

The guidance system for the rover’s six-axis robotic arm is key to its operation. This system allows the rover to detect the layout of plants in the chamber and to selectively monitor specific parts of individual plants. This capability is brought about by the “eye-in-hand” operation of the robotic arm, where the “eyes” are the cameras in the sensing unit that record in real time images of the robot’s surroundings, and the “hand” is the robotic arm.

The sensing unit includes an Odos 3D Time-of-Flight (ToF) camera (Milwaukee, Wisconsin, USA) and a Keyence LJ-V7300 2D in-line laser profilometer (Osaka, Japan). The long sensing range (0.5 to ~ 6 m) and wide field-of-view (43° × 33°) of the ToF camera allows for the creation of a rough (± 1 cm) 3D map of the growth chamber environment. Once a target plant is identified, the robotic arm approaches the plant and uses the laser profilometer to capture highly repeatable depth measurements within a relatively short measurement range.

#### Creation of the chamber-level map

**Fig. 3:**
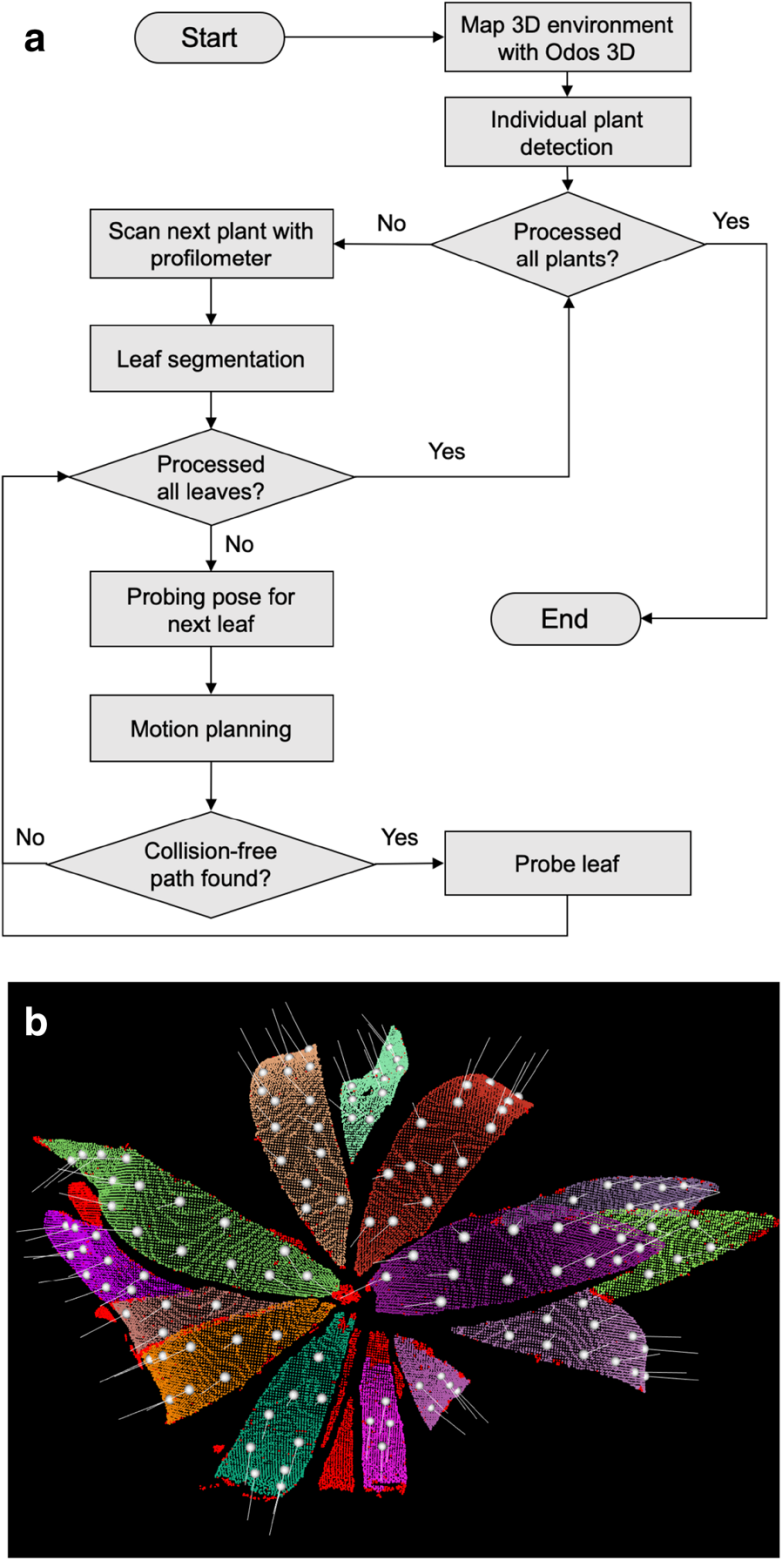
**a** Software pipeline for combining Odos 3D images with profilometer data to refine the leaf position map and obtain information to precisely position the leaf probe. **b** Leaf segmentation and probing location candidates. Image is a top view of the 3D point clouds obtained with the laser profilometer. Red points are rejected by 3D region growing segmentation. Any other color represents a different leaf segment. A feasible probing location is shown with a white sphere and the surface normal is shown as a grey line

After the rover enters a chamber, the Odos 3D camera rapidly creates a chamber-level environment map, which is refined by the profilometer’s high-resolution distance sensing capability. The 3D point cloud from the Odos camera is used to locate each plant and estimate its size and depth; however, depth sensing of the Odos camera is inaccurate and noisy. Therefore, the short-range 2D laser profilometer scans each plant from the top with a sweeping motion to create a higher precision map. The information from the two sensors is fed into a robotic leaf probing information pipeline to allow for precise motion control of the robotic arm (Fig. 3a). Accurate depth sensing and surface normal estimation of leaf position are needed not only to prevent the arm from colliding with plants as the sensors are positioned, but also to precisely position the fluorometer probe with respect to the leaf surface. The high-precision 3D scan from the profilometer enables accurate surface normal estimation. The surface normal leaf position is estimated based on principal component analysis (PCA) of the local neighborhood around a 3D point.

#### Leaf segmentation

Leaf segmentation is a critical step for the rover to identify leaves and the surfaces to probe on leaves. For most plants, leaf surfaces tend to be smooth, with neighboring points on the same leaf having similar surface normal direction and low curvature. Thus, to determine suitable probing locations, a segmentation algorithm was developed to extract 3D point clouds for the large, smooth portions of leaves. The algorithm used is based upon previous results, which showed that the 3D region growing segmentation algorithm with smoothness constraint effectively rejects noise and can accurately detect probing regions on plants [6]. In brief, piecewise smooth point clusters are extracted from the 3D point clouds to obtain leaf segments that are the large parts of plant leaves (Fig. 3b).

The number of acceptable leaf segments for probing depends on the geometry of the leaf surface. For instance, a soybean leaf is relatively small, flat, and round. Therefore a “leaf segment” is likely to be the whole leaf. For leaves such as maize leaves, the leaf is elongated and can be twisted. As such, multiple probing locations may be found on a single maize leaf. To accommodate different potential species of interest, the parameters of the 3D region growing segmentation can be adjusted so that leaf segments of various sizes can be produced [7].

#### Data acquisition workflow

During the course of an experiment, plant pots are maintained in the same position within a chamber. Their positions are automatically mapped during the first run of data acquisition. The rover is programmed to collect data in each chamber at scheduled times every day. For each plant, the rover executes a sequence of operations. First, the rover uses the plant height estimated in the last run to position the ToF camera to acquire an initial depth image. Then, plant height is updated and the imaging position for each camera is calculated. Next, the cameras are positioned such that they can all image an area of 0.5 × 0.5 m at the plant height. Subsequently, a high-precision 3D point cloud is acquired by rotating the 2D laser profilometer with the robot arm. Leaf segmentation is performed on this point cloud. Finally, fluorometer data and hyperspectral line scans are acquired on leaf surfaces if a collision-free path exists [7, 8].

### Sensors

#### Sensing unit

**Fig. 4:**
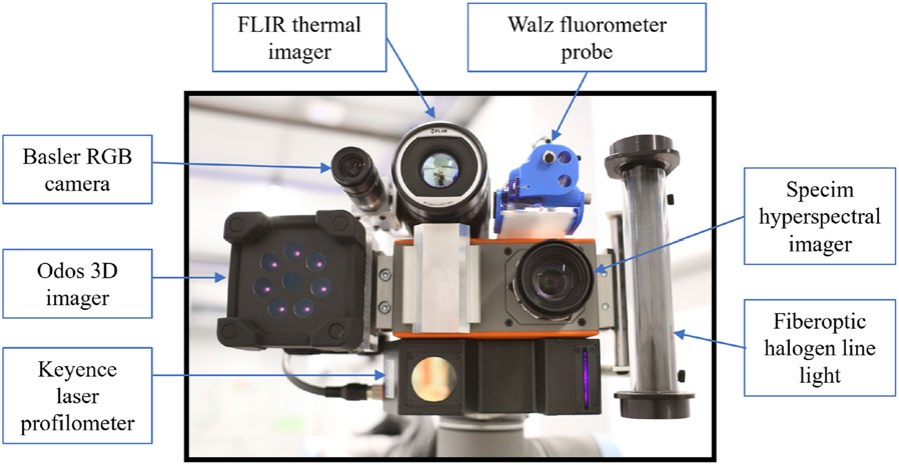
The sensing unit on the headpiece of the robotic manipulator

The sensing unit is equipped with various sensors to monitor the growth and physiological activities of plants in the chambers (Fig. 4). Current sensors include a Basler 5-megapixel RGB camera (Ahrensburg, Germany), a Keyence laser profilometer, an Odos 3D camera, a Specim VNIR hyperspectral camera (Oulu, Finland), and a Walz PAM fluorometer probe (Effeltrich, Germany). The output from the sensors is available to users as raw data or in processed form via custom web-based software tools (described in “Input instructions” and “Data management” sections below) developed for the Enviratron project. These tools allow users to browse the collected RGB, thermal, hyperspectral, and fluorometer data. The sampled data can be exported in a CSV or JSON text format, and synthetic images can be exported as RGB images in a PNG format. The hyperspectral reflectance plots can also be exported as PNG images.

#### Hyperspectral camera

Hyperspectral reflectance spectroscopy measures light reflectance patterns at different wavelengths extending through the range of photosynthetically active radiation (PAR, 400–700 nm) to near infrared (NIR, 700–900 nm) [9]. The rover is equipped with a Specim hyperspectral camera that is calibrated against a white sheet flipped down from a rod that projects from the sensing unit on the robotic arm. Two types of hyperspectral imaging are performed for each plant. One is top-view imaging under chamber lighting. Since the hyperspectral camera is a line-scan camera, it is rotated by the robot arm to capture a 2D image. The other type of imaging is leaf-tracing hyperspectral imaging under halogen line lighting on the sensing unit with the chamber lighting off. Each 3D leaf segment is dissected into 5 mm slices along its major axis. The hyperspectral camera is positioned to image slices at a distance of 0.25 m, with the imaging sensor scanning as parallel to the slice surface as possible.

**Fig. 5:**
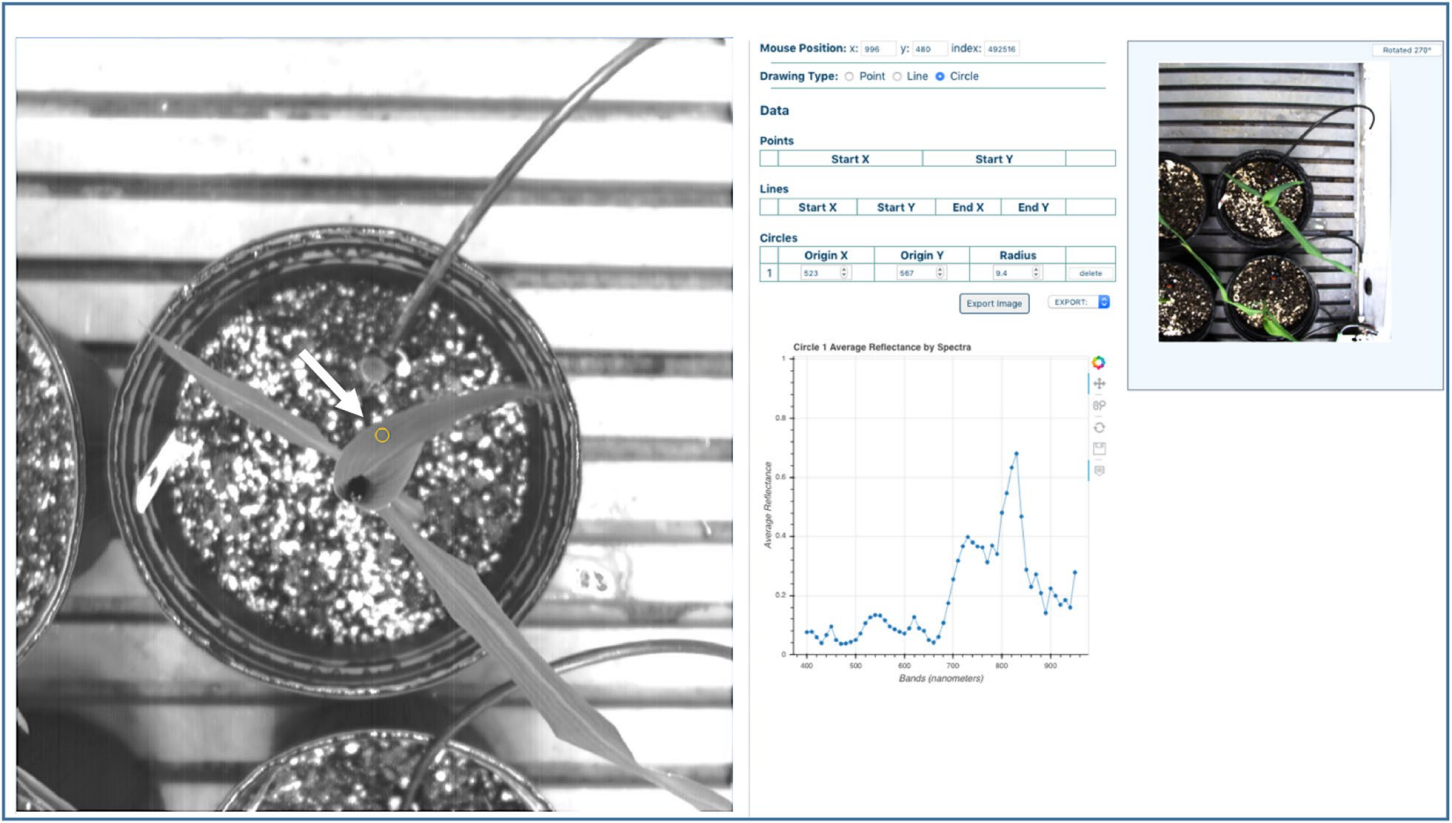
Output from the hyperspectral camera. Synthetic image (left) upon which users can draw shapes (see white arrow pointing to small circle) to obtain average reflectance spectra. Coordinates of the shape are shown on a table to the right of the central image. RGB image (upper right corner) is a reference image of the target plant

An example of the output from the hyperspectral camera is shown in Fig. 5. The user is presented with a synthetic image and an RGB image for comparison. The user chooses a line or circle on the synthetic image, which produces a plot of average reflectance across the 56 collected wavelength bands. Different materials have spectral signatures that correlate to different plant characteristics such as photosynthetic active biomass, pigment content and water status [10]. For example, the first derivative of the reflectance spectra in the red edge region (around 700–720 nm) has been used to assess chlorophyll indices [9, 11–13]. Other features can be extracted from a combination of single wavelengths. Kong et al. [14] used NIR hyperspectral imaging to determine the distribution of malondialdehyde, a stress indicator, in rape leaves. They found that an optimal prediction performance for malondialdehyde detection was achieved by an extreme learning machine model with 23 wavelengths selected by competitive adaptive reweighted sampling (CARS).

#### Fluorometer

A physiological parameter that the robotic rover can measure is intrinsic photochemical efficiency of light harvesting by photosystem II (PSII) using the pulse amplitude modulated (PAM) fluorometer [15]. The rover is equipped with a Mini-PAM-II fluorometer (Walz Heinz GmbH, Effeltrich, Germany) which measures fluorescence with a fiber-optic probe. The advantage of using the PAM-fluorometer in the Enviratron is that one can quantify changes in the fluorescence yield excited by the light pulses in a background of actinic and saturating lights [16].

**Fig. 6:**
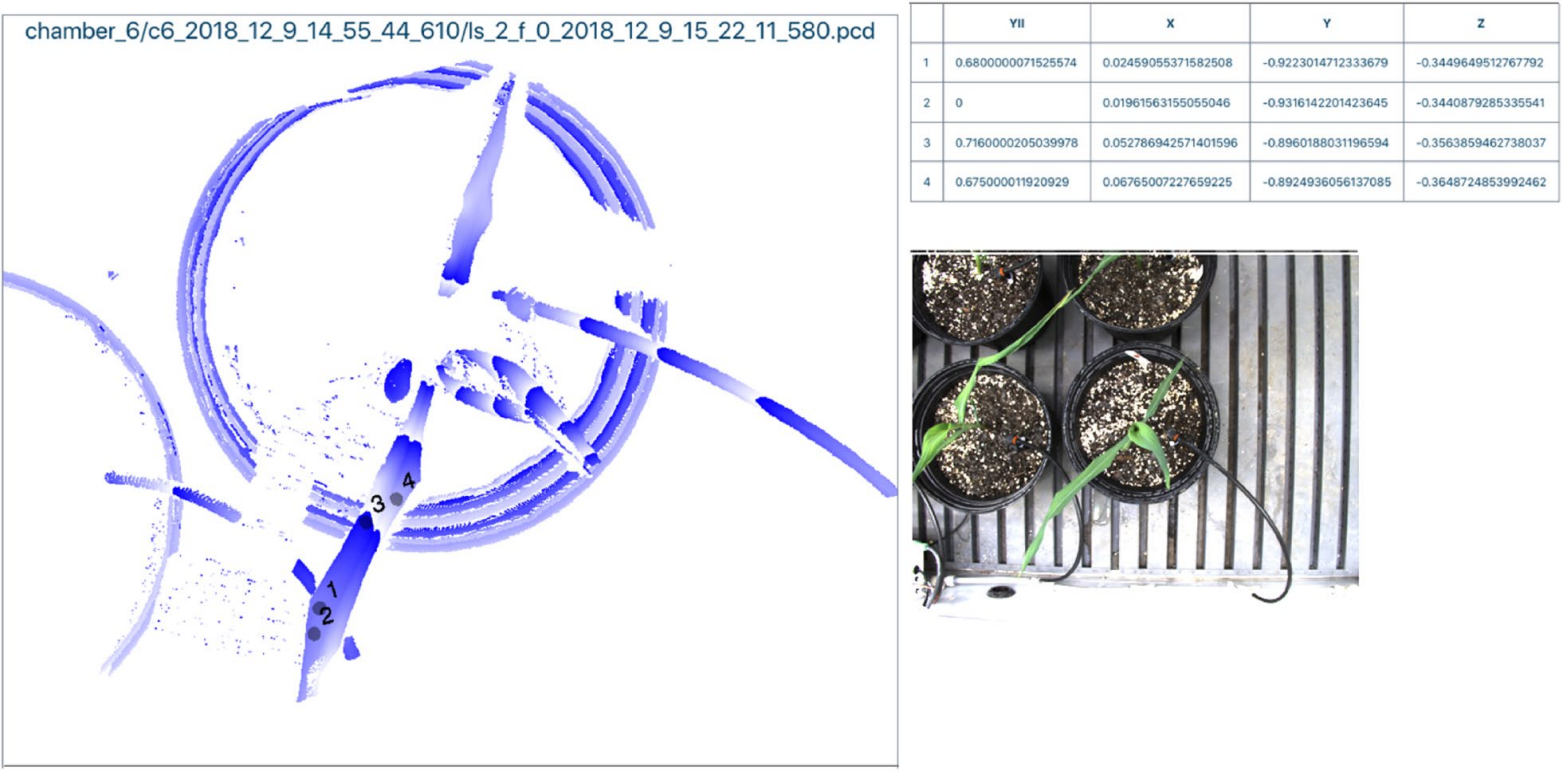
Output from the fluorometer probe. Synthetic image (left) shows four sites on the leaf where the fluorometer probe has taken a reading. Table to the right of the central image indicates the coordinates of the probe and the value at three of the sites for YII, an indicator of photosystem II photochemical activity. RGB image to the right is a reference image of the target plant

The Mini-PAM-II fluorometer is normally a portable device that positions its probe with a leaf clip about 8 mm from the leaf and at a 60° angle from the leaf surface. The robot arm operates without a leaf clip, but positions the fluorometer probe in a similar manner by extending it on a pneumatically actuated rod toward the leaf surface. At present the robot is not programmed to probe user specified places on a leaf, but instead the rover chooses even surfaces on the leaf from the segmentation analysis. However, the exact coordinates at which the measurements are taken are recorded and displayed on an image of the plant (Fig. 6).

The fluorometer data processed by the custom software developed for the Enviratron is presented as a 3D image of the subject plant with numbered markers representing the fluorometer measurement locations. For each fluorometer measurement, the value of YII and X, Y, Z coordinates are also presented in a table (Fig. 6). YII is the effective photochemical yield of PSII when measured in the light. YII = (F′_M_ − F)/F′_M_, where F′_M_ is the maximum fluorescence level of an illuminated sample [17]. F′_M_ is induced by a saturation pulse from the fluorometer’s laser which temporarily closes all PSII reaction centers and F is the momentary fluorescence level of an illuminated sample measured shortly before the application of the saturation pulse. PSII is particularly sensitive to reactive oxygen species (ROS) produced in plants by heat stress [18], and therefore, YII could also be used as a proxy for heat-induced ROS [19].

#### Thermal camera

**Fig. 7:**
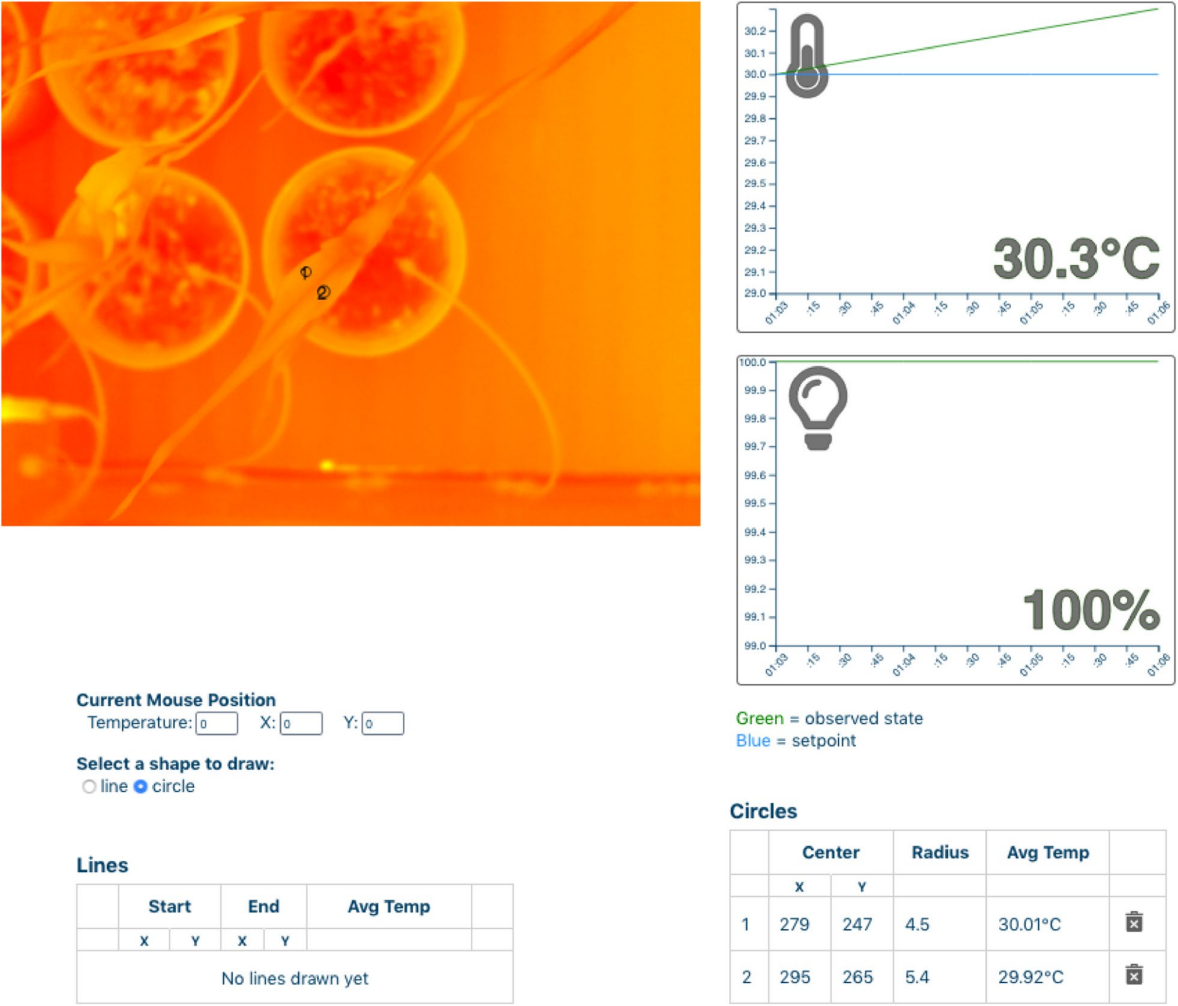
Thermal image of a target plant taken with IR camera. Users can draw a shape on the synthetic image (see two circles) and obtain the average temperature within that shape. Coordinates of the shape and the average temperature are given in the table below the image. Graphs to the right of the plant image show ongoing chamber temperature and light intensity changes

A FLIR A325sc thermal camera on the rover is used for thermographic imaging and analysis. As with the hyperspectral and fluorometer data, the user is presented with the processed thermal data as a synthetic image based on the source data. With the web-based tools, the user can sample the underlying data by drawing lines and circles on the synthetic images to obtain the average temperature within those shapes (Fig. 7). Leaf temperature readings have a number of uses including a means to estimate stomatal conductance. Stomatal conductance is an important feature to monitor in order to assess gas exchange and transpirational water loss, which are parameters important to crop growth and biomass production (for example, see [20]). Unfortunately, there is not a good way to measure stomatal conductance in a survey or high throughput mode. A number of studies have adopted thermography as a proxy for stomatal conductance measurements because leaf temperature varies with transpiration rate, which is largely a function of stomatal conductance [21–23]. For example, infrared thermography has been used on young wheat and barley seedlings to select genotypes capable of maintaining stomatal conductance in response to water deficit or osmotic stress associated with saline conditions [21, 24].

### Chambers

The plant growth chambers in the Enviratron were designed and built by Percival Scientific, Inc. (https://www.percival-scientific.com). The chambers are a modification of Model PGC-20L1 that has been adapted to accommodate a rover. The chambers are divided into two compartments, a plant growth compartment of ~ 1.8 m^2^ and a vestibule. The vestibule accommodates the rover and minimizes disturbance to the environment during entry and exit of the rover. The rover enters the vestibule through a sliding door that is activated when the rover approaches the chamber. The door can also be controlled through the chamber control system, or via manual override switches located inside and outside of the vestibule. The vestibule is separated from the plant growth compartment by a curtain that rolls up and down. The curtain is made of weatherproof fabric, one side of which has a reflective white surface. The white surface is positioned toward the growing space and reflects light from the LED canopy onto the plants when the curtain is down. Once the rover is inside the vestibule, the sliding door closes and the curtain is instructed to roll up, giving the rover access to the plants. Similar to the sliding door, the curtain can be actuated through the chamber control system, or via a manual position override switch located inside the vestibule.

For each growth chamber, Percival Scientific provides an http-based API (application programming interface) for monitoring and setting temperature, lighting, humidity, and volumetric water content as well as operating the doors and curtains and configuring/controlling other aspects of chamber operations. The API is a part of Percival’s IntellusUltra^®^ controller. In the case of monitor-only loops or values for relays that control doors, curtains, or lighting, it provides a rather direct link back and forth to the API. In other cases, the links are rather indirect as they form the setpoint for closed feedback loops that use the setpoint to control via tuned proportional-integral-derivative (PID) controllers on the chamber (such as with the temperatures, humidity, CO_2_, and volumetric water content), or they are part of alarm and safety conditions that the controller constantly monitors. The rover operates the doors, curtains and other aspects of the chambers via the custom software that uses this API. This software also schedules all environmental conditions in the chambers over the course of an experiment.

The chamber control system provides two communication pathways that facilitate remote monitoring and control functionality through Ethernet and Streaming Text Orientated Messaging Protocols (STOMPs). The Ethernet connection allows the rover and other external computational systems to remotely control air circulation dampers, vestibule curtain and door positions, lighting, temperature, humidity, CO_2_, and water delivery. The STOMP protocol feeds to a database where long-run experimental data may be more easily accessed. As a redundancy, the controller on each chamber retains up 1 year of data on a microSD card. Each minute, the controller logs set points, process values, digital I/O states, alarm conditions and operating modes.

#### Lighting

Light quality in controlled environment systems can have profound effects on plant phenotypes [25–30]. Therefore, the lighting system in the Enviratron plant growth chambers controls light intensity and light quality for an array of spectrum-specific, SciBrite™ 7-color LED modules. In addition to their highly ‘tunable’ architecture, the LEDs use less energy and produce less heat than conventional light sources used in other plant growth chambers. An advantage of using LEDs is that the lights can be rapidly cycled off and on without a warm-up period. This allows for better integration with the rover’s schedule and additional capacity to optimize data acquisition timing and duration. Lastly, LEDs benefit from greater stability (i.e., less performance decay) over their rated life and longer operational lifespans than conventional fluorescent or high intensity discharge (HID) bulbs in controlled environment applications.

**Fig. 8:**
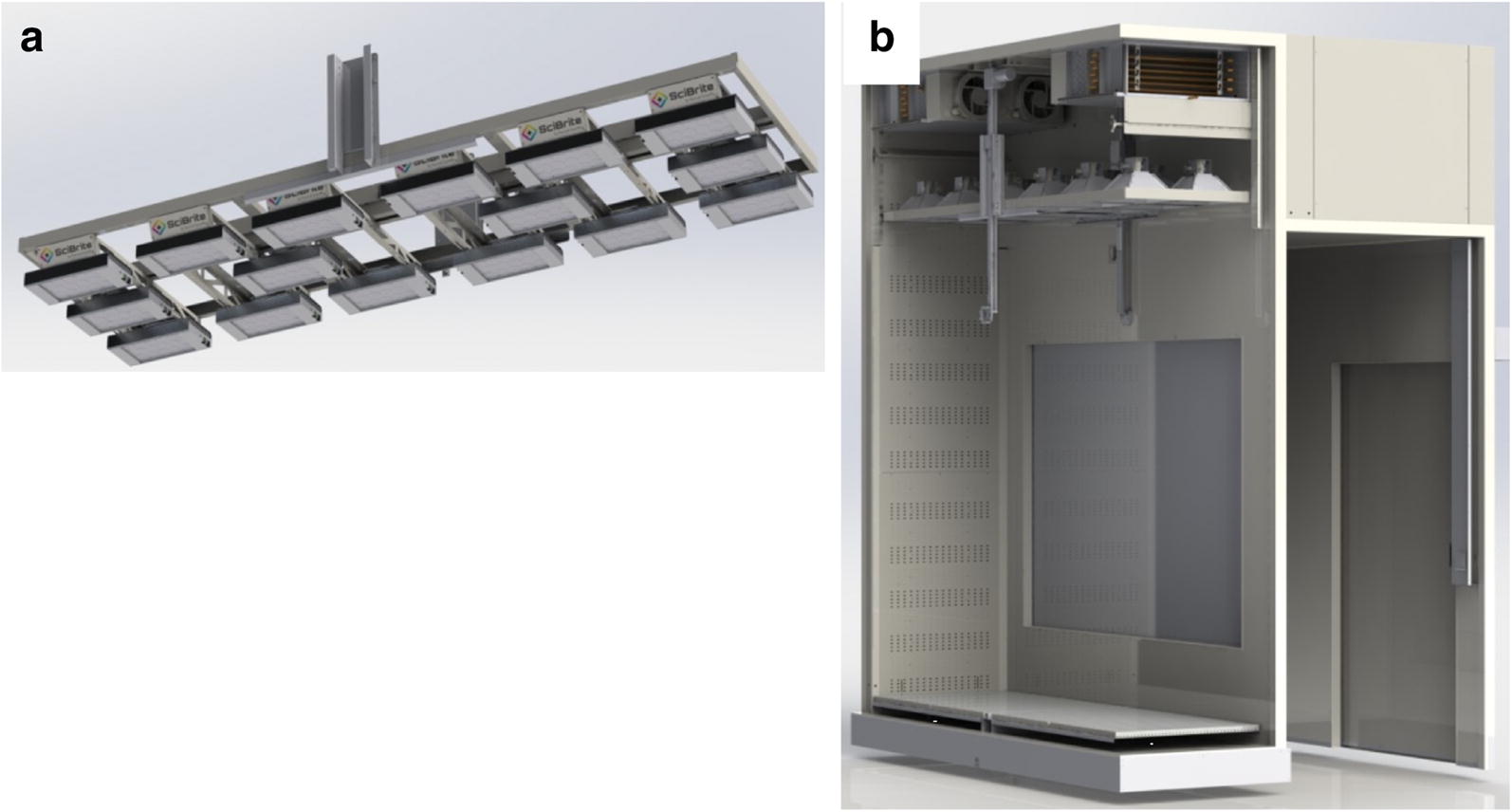
Plant growth chamber lighting. **a** Rendering of vertically adjustable LED lamp canopy. **b** Cutaway view of PGC-20L1V2 plant growth chamber with canopy at maximum height. Plant growth compartment and vestibule are shown

The LED canopy in each chamber is composed of an array of sixteen 7-color SciBrite LED modules (Fig. 8a). The canopy is vertically adjustable via actuators controlled by a position switch integrated into each chamber’s control panel (Fig. 8b). Each SciBrite LED module consists of multiple clusters of LEDs, with each of the individual 7 colors within each cluster (Table 1). This ensures an even distribution of colors across each module, which results in color homogeneity at the plant canopy. Each color is independently dimmable through a dedicated output channel on the control system as a percentage of total output. Two of the colors are broad-spectrum, white LEDs, and the other five produce much more narrowly focused light (Additional file 1: Fig. S1). The modules are capped by lenses to increase intensity and light uniformity at larger distances (Additional file 1: Fig. S2). The LED lighting system inside each plant growth chamber is capable of producing an average total irradiance of 1205 µmol/m^2^/sec at 60 cm from the canopy with a coefficient of variation of 0.13.

**Table 1:** Spectral output from the SciBrite LED modules

LED type	Spectral output	Percent of total output
Cool white	6000 K	22
Warm white	3000 K	22
Royal blue	451 nm	14
Green	520–535 nm	6
Red	620–630 nm	12
Deep red	650–670 nm	16
Far red	720–740 nm	9

#### Temperature and humidity

The chambers can control temperature in a range of 10°–44 °C with a uniformity of ± 1 °C within the plant growth compartment. Uniformity of these parameters are extremely important as they can greatly influence plant performance [31]. Chamber temperatures vary by no more ± 0.5 °C and in the trace shown here the fluctuations vary by no more than ± 0.4 °C (Additional file 1: Fig. S2). Relative humidity (RH) can be controlled down to 40% ± 5% between 15 and 30 °C. Humidification is provided by spray nozzles under the floor diffuser panels, while dehumidification consists of electrical heaters and a dehumidifying evaporator. Humidity levels are sensed by an advanced electronic RH sensor with a measurement range 0–100% RH ± 3%.

#### Airflow

The modified chambers are configured with vertical airflow through diffusers in the floor and side-walls. The floor diffusers allow for a more even, vertical airflow pattern, as well as ensuring that the mist from the nozzles spreads throughout the rest of the chamber, while the side-wall channels create additional heat uniformity. Computational fluid dynamics (CFD) models were run to ensure specified temperature uniformity requirements, without allowing airspeeds to rise above 0.25 m/s. There is a delicate balance in controlling airspeeds as too little airflow will not maintain temperature uniformity, whereas too much airflow can produce movement or touch responses in plants [32–34]. There are dampers inside the sidewalls of the chambers to temporarily cut off airflow to minimize leaf flutter even further while the rover is imaging plants in the chamber. The Enviratron software activates/deactivates the dampers whenever the Rover opens or closes the chamber curtain.

#### Irrigation

Water delivery is managed in the chambers via a closed-loop drip irrigation system. The volumetric water content (%VWC) of the soil is measured using an EC-5 Decagon sensor (METER Group, Inc.) that measures the dielectric constant of the soil using capacitance/frequency domain technology. Its 70 MHz frequency minimizes salinity and textural effects, making this sensor accurate in almost any soil or soilless media. The controller regulates the release of water to each pot in an additive-only closed-loop feedback system via a series of irrigation drippers. At any moment, if the %VWC of the sample pot is lower than the desired level, the controller adjusts the amount of water released into the pots, and rechecks the new value against the input. When the %VWC level reaches the set point in the sample pot, the system shuts off the valve. By branching each irrigation dripper line off of a single supply loop, each dripper maintains approximately equal water pressure and water delivery to each line. The chambers in the Enviratron are served by a common deionized water supply to which liquid fertilizer can be added by Dosatron injector (Clearwater FL) according to user instructions. The common fertilization system can be overridden for manual fertilization if, for example, a user wants to test the effects of different plant nutrients.

#### CO_2_ enrichment and depletion

Plants respond to CO_2_ concentration at molecular, cellular, biochemical and physiological levels (see for example [35]). CO_2_ levels in the chambers are measured via a Vaisala GMP252 sensor. CO_2_ concentrations can be controlled between 150 PPM (the typical level for carbon dioxide starvation studies [36]) to 5000 PPM (the sensor’s saturation limit) with an accuracy of ± 10% (a limitation of the sensor, [37]). The system uses a PID feedback loop in which signals from the sensor are the input. When the current level of CO_2_ is lower than the chamber’s set point, a solenoid valve is activated to slowly release CO_2_ into the chamber. Likewise, when the CO_2_ concentration is higher than the chamber’s set point, a fan is activated to draw air through several layers of sodasorb (a mixture of calcium hydroxide, lime, sodium hydroxide, water, and trace amounts of potassium hydroxide) to remove CO_2_. The fan and solenoid flow-rates are tuned such that CO_2_ can be added at a rate to balance the slower CO_2_ depletion by the CO_2_ removal system.

### Experimental design

Experimental design is particularly important for the Enviratron due to the limited growth space in each chamber. The configuration of the Enviratron system naturally leads to split-plot experimental designs [38]. The eight growth chambers function as whole-plot experimental units to which levels of environmental treatment factors can be randomly assigned. Inside each chamber, pots serve as split-plot experimental units to which levels of split-plot treatment factors may be assigned. As an example, consider an experiment to study the effects of temperature, humidity, and CO_2_ level on the growth and development of 14 plant genotypes. For simplicity, suppose there are two temperatures (low vs. high), two humidity regimes (low vs. high), and two CO_2_ levels (low vs. high). The eight combinations of temperature, humidity, and CO_2_ level are randomly assigned to the eight Enviratron chambers. Within each chamber, the 14 genotypes are randomly assigned to pots (pictured as circles in Fig. 1a). The robotic rover can then generate multivariate repeated-measures data on these 8 × 14 = 112 plants throughout a monitoring period of interest.

Although this data collection process will produce a large dataset, the experiment would be unreplicated at the whole-plot level because there is only one experimental unit (chamber) for each combination of temperature, humidity, and CO_2_ levels. To obtain the replication necessary for making inferences about the effects of temperature, humidity, and CO_2_ level, the data collection process must be repeated multiple times with different sets of plants and new random assignments of environmental conditions to chambers and genotypes to pot positions within each chamber for each replication.

If more than eight environmental settings are of interest, an incomplete block design, with multiple Enviratron runs as blocks, can be used to compare all the environmental settings of interest. If fewer than eight environmental conditions are of interest, replication can be obtained with one set of plants during a single monitoring period. For example, if the focus is on low vs. high temperature effects with other environmental factors held constant, four of the Enviratron chambers can be randomly selected for the low temperature setting and four for the high temperature setting. This would allow for the evaluation of temperature effects, genotype effects, and temperature-by-genotype interactions using data from a single Enviratron run.

The Enviratron can also be used to execute response surface designs intended to identify optimal conditions for plant growth. As a simple example, suppose the temperatures 22, 24, 26, and 28 °C are randomly assigned to the eight growth chambers, with two chambers per temperature and all other environmental factors held fixed across chambers. With 14 genotypes randomly assigned to pot positions within each chamber, a model can be fit that allows the expected value of a response variable of interest to change according to a genotype-specific quadratic relationship with temperature. Based on the model fit, an estimate can be made, separately for each genotype, for the temperature in the range 22 to 28 °C at which the expected value of the response variable is maximized.

## Discussion

The Enviratron is a unique phenotyping platform for studying plant performance under different controlled environmental conditions. The facility differs from most other phenotyping facilities by (A) offering multiple environments to test plant performance, and (B) bringing the sensing equipment to the plants, allowing them to stay in their growth environment throughout an experiment. Under these conditions, crop plants, such as maize, can be grown to full height and maturity, enabling automated multi-climate life-cycle analysis. To monitor plant performance in these multiple environments, a sensor-equipped robot circulates in the Enviratron among an array of growth chambers. The robot non-destructively monitors plant performance by imaging the plants and positioning probes within millimeters of the leaf surface to measure properties such as PAM-fluorescence.

The growth of plants under controlled environmental conditions allows for an investigator to define specific environmental conditions and determine the effects by modifying those conditions [39], thereby promoting reproducibility in phenotype observations [40]. Field phenotyping is beset by high spatial and temporal heterogeneities and fails to provide the opportunity to reproduce experiments under the same conditions [41]. However, controlled environment experiments can be problematic in that results are often difficult to relate to or translate directly into yield performance under field conditions [41]. Nonetheless, advances in the design of growth chamber experiments and growth chamber control technologies, such as those outlined in this report, can provide reproducible assessments of the effects of environmental conditions on plant performance.

Various experiments have been planned or beta-tested in the Enviratron. One type of experiment has involved incrementing a single environmental condition from chamber to chamber, such as temperature to test how plants respond and tolerant increasing temperatures. The environmental variables that can be tested include temperature, soil water content, humidity, light intensity or quality, photoperiod, CO_2_ levels, soil nutrients and so forth. Other experiments could involve testing many different environmental variables at once, as in the case of simulating climates in different parts of the world. The system has a graphical interface for inputting variables with smooth transitions, which can be controlled diurnally or seasonally for longer term experiments. An important feature of the system is that the timing of periodic processes such as photoperiod can be offset from chamber to chamber so that the rover monitors the plants in different chambers at the same time of virtual day.

As pointed out above, a major limitation of the Enviratron is the lack of experimental space to test many genotypes. However, if one intends to test the effects of different environmental conditions on the performance of limited number of selected genotypes, then the Enviratron can be an extremely valuable resource.

## Conclusions

Assessing plant performance under different environmental conditions is of utmost importance in the face of climate change. The Enviratron offers the opportunity to analyze plant performance under different environmental conditions. The Enviratron is composed of an array of growth chambers with a roving robot that operates on a sensor-to-plant principle. This mode of operation allows for plants to be monitored in situ and for precision positioning of plant sensors by the robot.

## Methods

### Input instructions and data management

**Fig. 9:**
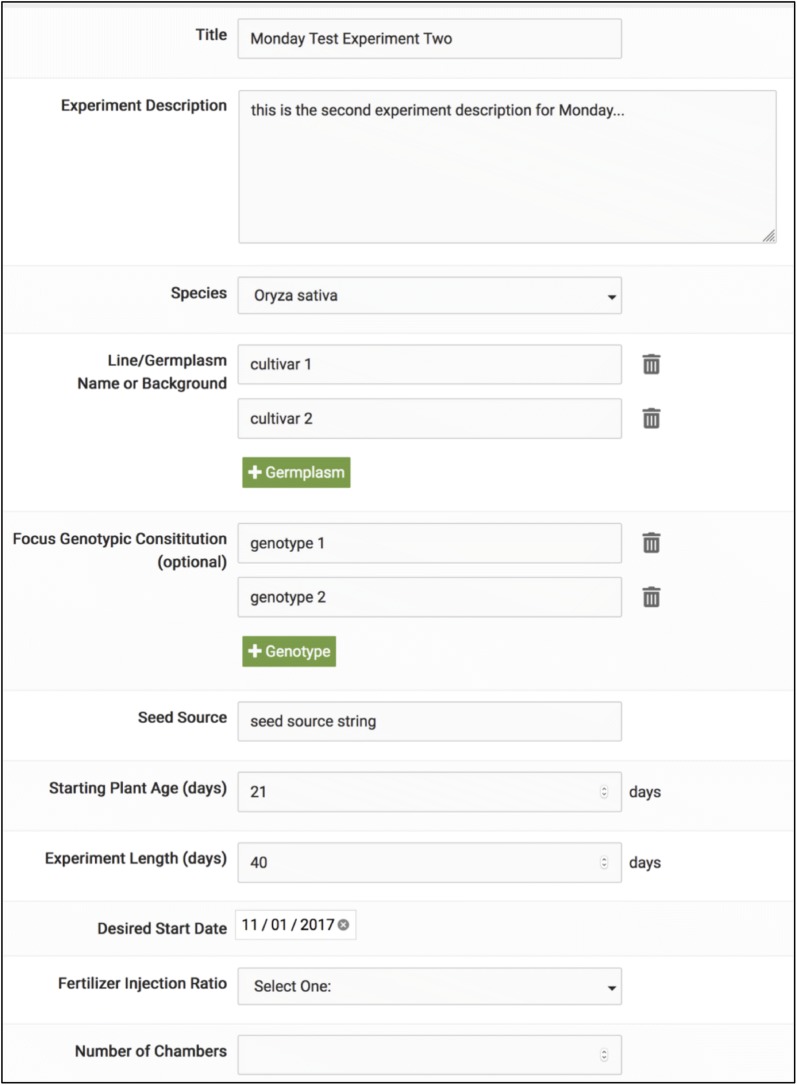
User provided metadata for an Enviratron experiment. User describes experiment and inputs instructions for operation of the chambers and the rover. Shown is a screenshot of the front page of the application for inputting metadata and instructions

User instructions for controlling the growth chambers and operating the rover are entered via a web-based application. The app is intended to help the user meet “Minimum Information About a Plant Phenotyping Experiment” (MIAPPE) standards for reporting their experiment [37]. The queries in the application concern a list of attributes that provides a useful description of the experimental plan for a plant phenotyping experiment and an understanding of the data obtained in it. Each page of the app aggregates attributes detailing specific aspects of an experiment that are important to report. The front page of the app is shown in Fig. 9. A git repository containing code developed to operate and support the Enviratron is available online at https://gitlab.com/dill_picl/enviratron.

Experimental parameters are stored in a SQL RDMS (Relational Database Management System). Stored data includes experimental design metadata about the experiment (species, genotypes, germplasm, experiment length, etc.), environmental conditions within the growth chambers, and observations/measurements from the rover. Chamber environmental data include temperature, lighting, RH, fertilizer ratios, and watering as well as expression values and parameters for statistical tests. In addition to the programmed environmental data, actual observed environmental condition data are also available. The metadata are represented using biological ontologies and accepted standard terms where possible.

Once an experiment has concluded, data generated by the Enviratron are stored and made accessible in various ways. Each research group may wish to store and analyze data in-house using their own computer or they may want to store and share raw and/or analyzed data via their own library resources. Currently, the Enviratron system is designed to work as follows: Experimental design information for planning projects are represented via the CyVerse Discovery Environment. Data collected by the Enviratron rover are stored locally then copied to and made accessible via the CyVerse DataStore. CyVerse authentication services ensure data access integrity, and the DataStore provides sufficient capacity for long-term storage and DOI creation to support general access to large volume datasets. To enable generated datasets to be stored long-term in community repositories, the data need to be FAIR (findable, accessible, interoperable, and reusable). Toward that goal, Enviratron datasets are tagged with ontology-based meta-data terms, enabling the representation and use of these datasets via a number of repositories that are only now beginning to consider serving phenomics data.

Ideally, generated resources appropriate to share with colleagues will be moved into long-term community repositories such as MaizeGDB, SoyBase, Gramene, and others. For phenomics data, some repositories are only now coming online with the most up-to-date resources anticipated to be listed at http://nappn.plant-phenotyping.org/.

## Supplementary information

**Additional file 1: Fig. S1.** Relative intensity spectrographs of the seven colors produced by the SciBrite LED modules. The spectra were measured at 60 cm from an individual LED panel using an Apogee model SS-110 spectroradiometer. A smoothing filter was applied which effectively averages the nearest ± 2 nm wavelengths. Some noise is apparent in the green and far red LEDs because the overall intensities of these LEDs are lower compared to other LEDs. **Fig. S2.** Temperature uniformity across the plant growth compartment. The uniformity along an entire shelf or horizontal plane within the chamber is ± 0.5 °C. This is shown in the following graph as being within ± 0.4 °C (half of the full range of ∆°C = 0.8 °C). The test was performed with NIST-traceable Madgetech temperature sensors along the floor of the chamber with the chamber set to 30 °C for 8 h.

## Data Availability

A Git repository containing code developed for the Enviratron is available online at https://gitlab.com/dill_picl/enviratron. The facility will be operated as a fee-for-service facility by the Office of Biotechnology at Iowa State University. Questions and queries about use of the Enviratron facility should be directed to the Office of Biotechnology via biotech@iastate.edu.

## Abbreviations

2D: two dimensional
3D: three dimensional
API: application programming interface
CARS: competitive adaptive reweighted sampling
CFD: computational fluid dynamics
CSV: comma separated values
F: fluorescence level
F’_M_: maximum fluorescence level of an illuminated sample
FAIR: findable, accessible, interoperable, and reusable
FLIR: forward looking infrared
HID: high intensity discharge
IR: infrared
JSON: javascript object notation
LED: light emitting diode
MaizeGDB: maize genome data base
MIAPPE: minimum information about a plant phenotyping experiment
NIR: near infrared
NIST: National Institute of Standards and Technology
PAM: pulse-amplitude modulated
PAR: photosynthetically active radiation
PCA: principal component analysis
PDI: proportional-integral-derivative
PNG: portable network graphics
PPM: parts per million
PSII: photosystem II
RDMS: relational database management system
RGB: red, green, blue
RH: relative humidity
ROS: reactive oxygen species
SD: secure digital
SQL: structured query language
STOMP: streaming text orientated messaging protocols
ToF: time-of-flight
UGV: unmanned ground vehicle
VNIR: visible and near infrared
VWC: volumetric water content
YII: photochemical yield of PSII
