# Supplementary figures and images for: Assessing plant performance in the Enviratron
Source: Plant Methods. 2019 Oct 23;15:117. doi: 10.1186/s13007-019-0504-y (PMC6806530; doi:10.1186/s13007-019-0504-y)

## Slide 1
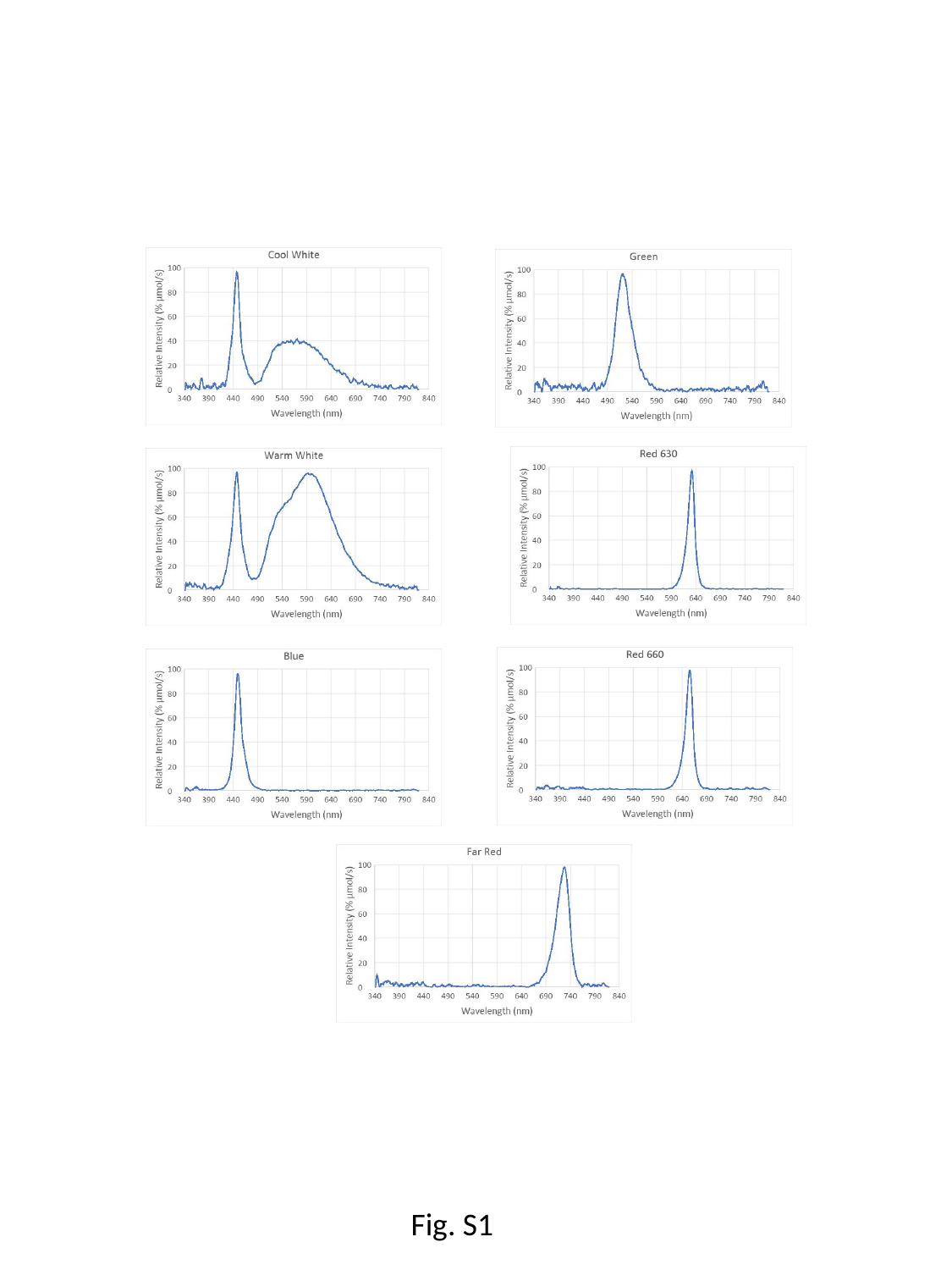

Fig. S1

## Slide 2
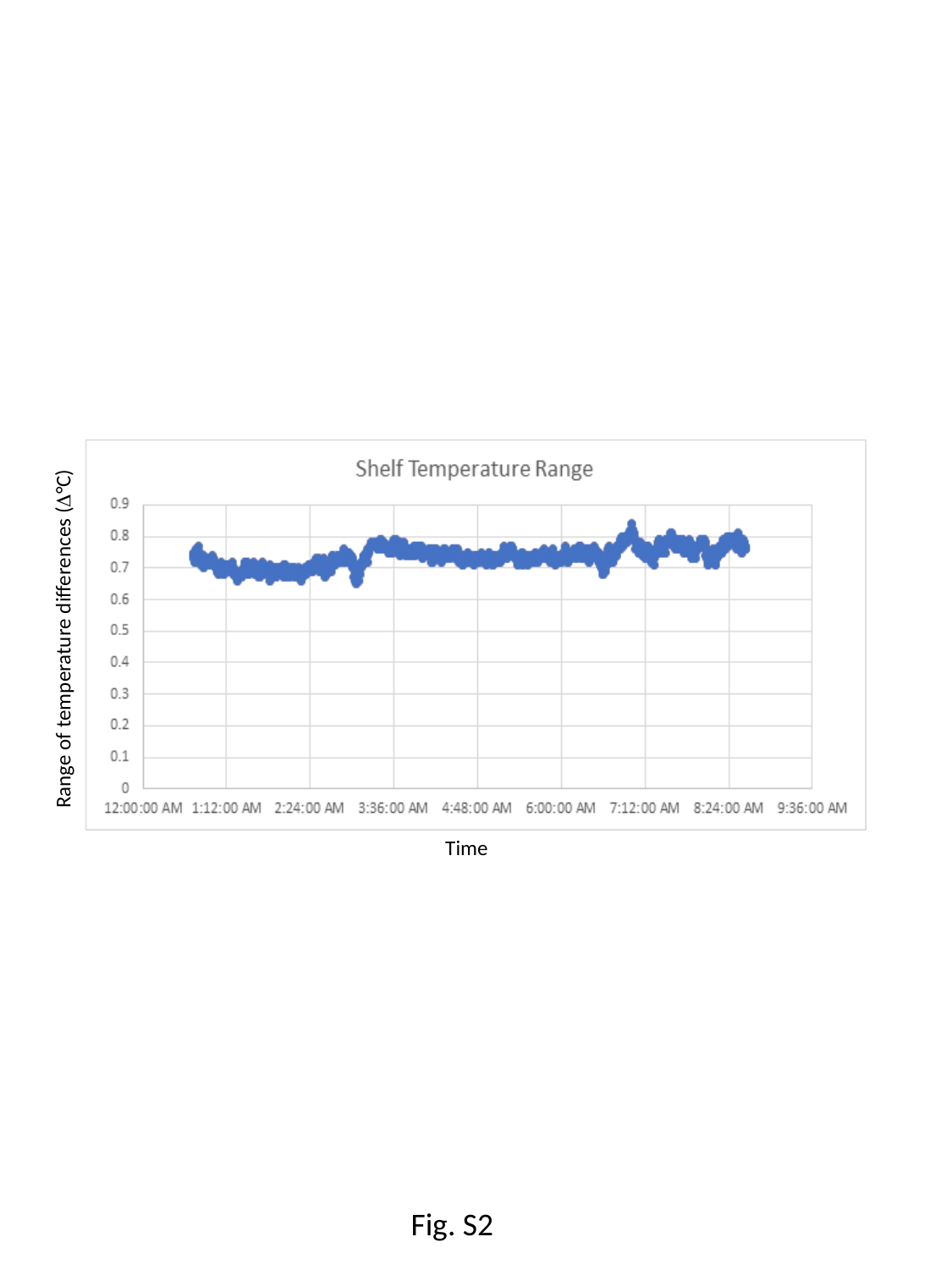

Range of temperature differences (D°C)
Time
Fig. S2

Supplement: Supplementary file 1 — Additional file 1: Fig. S1. Relative intensity spectrographs of the seven colors produced by the SciBrite LED modules. The spectra were measured at 60 cm from an individual LED panel using an Apogee model SS-110 spectroradiometer. A smoothing filter was applied which effectively averages the nearest ± 2 nm wavelengths. Some noise is apparent in the green and far red LEDs because the overall intensities of these LEDs are lower compared to other LEDs. Fig. S2. Temperature uniformity across the plant growth compartment. The uniformity along an entire shelf or horizontal plane within the chamber is ± 0.5 °C. This is shown in the following graph as being within ± 0.4 °C (half of the full range of ∆°C = 0.8 °C). The test was performed with NIST-traceable Madgetech temperature sensors along the floor of the chamber with the chamber set to 30 °C for 8 h. [file 13007_2019_504_MOESM1_ESM.pptx]
